# CusVarDB: A tool for building customized sample-specific variant protein database from next-generation sequencing datasets

**DOI:** 10.12688/f1000research.23214.2

**Published:** 2020-11-16

**Authors:** Sandeep Kasaragod, Varshasnata Mohanty, Ankur Tyagi, Santosh Kumar Behera, Arun H. Patil, Sneha M. Pinto, T. S. Keshava Prasad, Prashant Kumar Modi, Harsha Gowda

**Affiliations:** 1 Center for Systems Biology and Molecular Medicine, Yenepoya Research Centre, Yenepoya (Deemed to be University), Mangalore, 575018, India; 2 Institute of Bioinformatics, International Technology Park, Bangalore, 560066, India

**Keywords:** Next-generation sequencing, Variant protein database, NGS-pipeline

## Abstract

Cancer genome sequencing studies have revealed a number of variants in coding regions of several genes. Some of these coding variants play an important role in activating specific pathways that drive proliferation. Coding variants present on cancer cell surfaces by the major histocompatibility complex serve as neo-antigens and result in immune activation. The success of immune therapy in patients is attributed to neo-antigen load on cancer cell surfaces. However, which coding variants are expressed at the protein level can’t be predicted based on genomic data. Complementing genomic data with proteomic data can potentially reveal coding variants that are expressed at the protein level. However, identification of variant peptides using mass spectrometry data is still a challenging task due to the lack of an appropriate tool that integrates genomic and proteomic data analysis pipelines. To overcome this problem, and for the ease of the biologists, we have developed a graphical user interface (GUI)-based tool called CusVarDB. We integrated variant calling pipeline to generate sample-specific variant protein database from next-generation sequencing datasets. We validated the tool with triple negative breast cancer cell line datasets and identified 423, 408, 386 and 361 variant peptides from BT474, MDMAB157, MFM223 and HCC38 datasets, respectively.

## Introduction

Cancer genome sequencing projects have revealed thousands of genomic variations in cancers ( Forbes *et al*., 2010; Tate *et al*., 2019; Tomczak *et al.,* 2015; Zhang *et al.,* 2011). Some mutants are oncogenic and drive proliferation in various cancers. For example, amino acid substitution of arginine to leucine at position 858 (L858R) in the epidermal growth factor receptor (EGFR) gene has been observed in 17% of pulmonary adenocarcinoma patients ( Morgensztern *et al*., 2015). In addition, a mutation in gene BRAF V600E is known to drive some melanomas ( Chapman *et al*., 2011). Some of these mutant proteins are proteolytically processed in cancer cells, resulting in major histocompatibility complex (MHC) presentation of mutant peptides. These mutant peptides serve as neo-antigens that recruit T cells and result in immune activation ( Kreiter *et al*., 2015). However, not all mutants are encoded at the protein level. Therefore, it is important to identify mutant proteins expressed by cancer cells. However, there are no easy-to-use pipelines for biologists to identify such coding variants, which alter the protein sequences and may play an important role in tumorigenesis. Detection of cancer-specific proteoforms has been studied by several research teams using proteogenomics methods. This approach integrates proteomics data with genome sequence data to identify protein complement of genomic variants ( Menschaert & Fenyö, 2017; Nesvizhskii, 2014; Ruggles & Fenyö, 2016). Detection of coding variants can provide candidate molecules for novel therapeutic interventions ( Subbannayya *et al*., 2016).

Several tools have been developed in the last decade to carry out onco-proteogenomics. CPTAC ( Ellis *et al*., 2013) program was initiated to understand the complexity of cancer and its sub types using multi omics approach. Various tools and approaches have been employed to identify mutant peptides in cancers ( Mathivanan *et al*., 2012) ( Yeom *et al*., 2016) ( Nagaraj *et al*., 2015). Several qualitative and quantitative proteomics studies have been reported, which identify altered expression of proteins in cancers ( Subbannayya *et al*., 2015). Often, conventional workflows were used in such investigations, wherein a reference protein database was used to search tandem mass spectrometry data for identification and quantification of proteins ( Kelkar *et al.,* 2014). However, such a reference database is usually devoid of sample-specific amino acid variations resulting from genomic alterations. The publicly available databases such as dbSNP ( Sherry *et al*., 2001), COSMIC ( Forbes *et al*., 2015) and UniProt ( Apweiler *et al*., 2004) can be used to identify the variant peptides ( Alfaro *et al*., 2017) but millions of protein-variants from these databases might increase the probability of identifying false positives. Therefore, there is a need for an improved method to identify sample specific sequence variations at the proteomic level. Tools such as CustomProDB ( Wang & Zhang, 2013) and MZVar ( https://bitbucket.org/sib-pig/mzvar-public/src/master/) have been developed for generating variant protein database. These tools require processed files such as VCF or BED file as an input. Executing the preprocessing steps requires knowledge of computation and also requires multiple tools to generate the output. Hence, we developed CusVarDB with an in-built pipeline for genomics suite to identify variants and create custom variant protein database.

## Methods

### Implementation

CusVarDB is available at https://sourceforge.net/projects/cusvardb/ and http://bioinfo-tools.com/Downloads/CusVarDB/V1.0.0/. Our tool supports graphical user interface (GUI) for easy execution of next-generation sequencing (NGS) pipelines. The GUI was developed using Microsoft Visual Studio Community edition 2017. Linux commands were executed through the Python program (terminal.py) developed by Linwei available on GitHub. Portable version of Perl was used to execute the Annovar scripts. A python script was used to generate the custom variant database; the scripts were made portable using PyInstaller. The dry-run concept is implemented in the tool to customize commands according to user’s need and run in batches.

**Figure 1.**
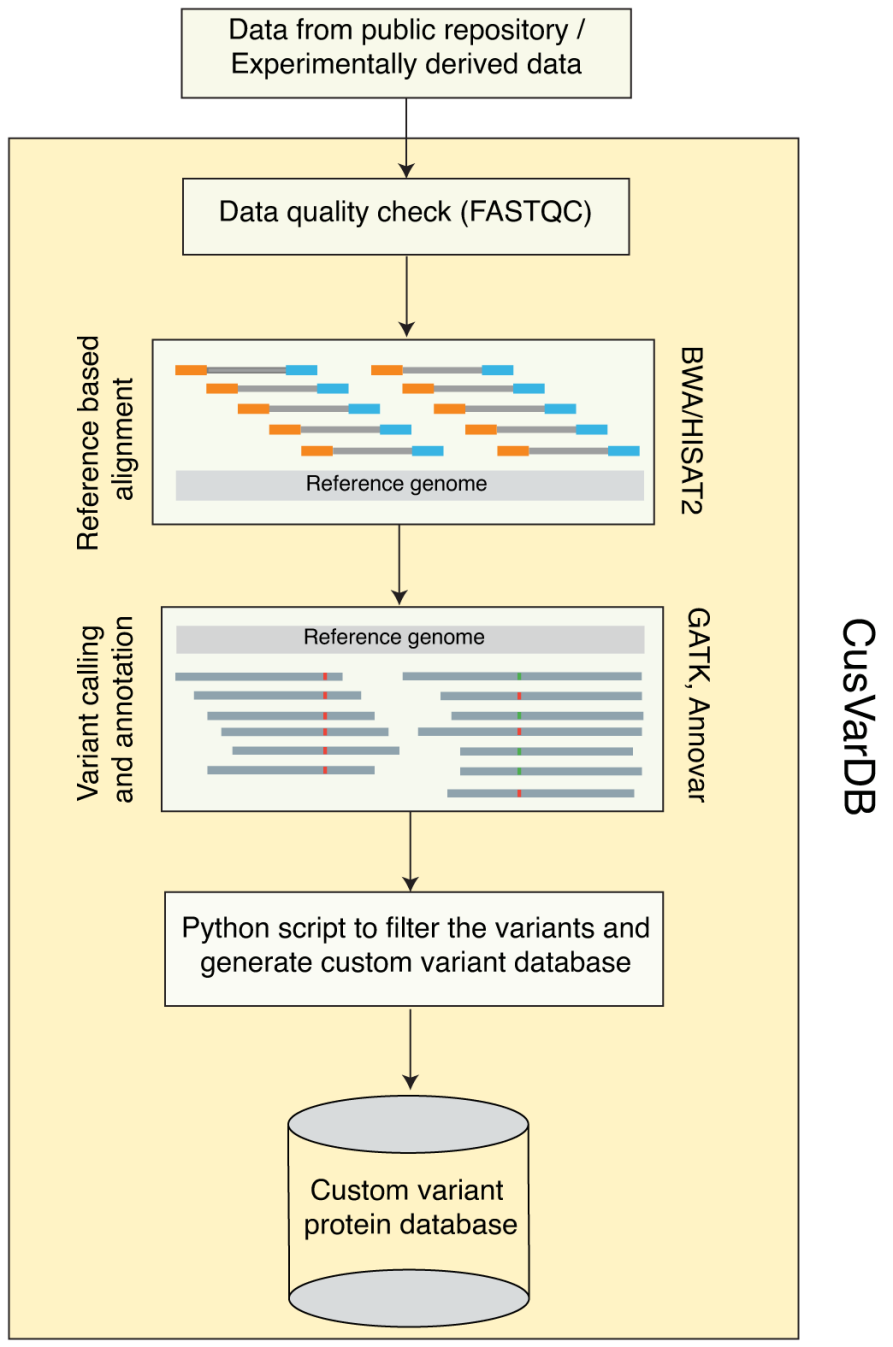
Schematic representation of the workflow.

CusVarDB inherits different NGS pipelines for genomics, RNA-Seq and exome-seq datasets. The tool performs the following steps: i) alignment of genomic data to a reference genome; ii) variant calling; iii) variant annotation; and iv) generate variant protein database ( Figure 1). Burrows-Wheeler Aligner (BWA) is executed for alignment of genome and exome ( Li & Durbin, 2009) data, while HISAT2 is used for RNA-Seq data ( Kim *et al*., 2015). Subsequent steps involving sample labelling, variant calling and annotation are performed using Picard, GATK ( McKenna *et al*., 2010) and ANNOVAR ( Wang *et al*., 2010), respectively. CusVarDB substitutes the amino acid variations from the previous steps to generate a custom variant protein database.

### Operation

CusVarDB runs on Windows 10 system. It requires Linux Bash Shell and ANNOVAR tool to be enabled and installed by the user. Minimum system requirements include Intel i5 or i7, having at least four cores with 8 GB of RAM and 1 TB hard drive. High performance processors such as Intel i9 or Xeon and large quantity of RAM can enable faster execution of tasks.

The tool requires FASTA, FASTQ and dbSNP database as input files. FASTA file will be loaded at configuration panel and indexed using bwa index or hisat2-build command. The quality control panel loads the FASTQ file and produces a quality check report using the FastQC tool. The third panel asks the user to load the FASTQ file(s), set the number of threads and RAM to perform the alignment and variant calling steps. This step produces the raw VCF file, which will be used for annotation in annotation tab. The annotation panel annotates the variants and incorporates those variants to the protein database to create a custom variant protein database. Detailed information of tool installation and execution of a test dataset are available in the manual (Manual.pdf) available to download here and on Zenodo ( Kasaragod *et al*., 2020b).

### Use cases methods

The genomics datasets downloaded from the SRA repository were subjected to a quality check using FastQC tool. Poor quality reads with Phred score less than twenty were trimmed using fastx_trimmer. Trimmed reads were mapped to human reference genome version 19 (hg19). AddOrReplaceReadGroup and Markduplicates operations from GATK were performed to add label information and to remove polymerase chain reaction (PCR) artefacts. Further, post alignment processing steps such as IndelRealigner and BaseRecalibrator were performed to correct the mapping errors made by alignment tool to increase variant calling accuracy. Variant calling was performed using HaplotypeCaller. Finally, raw variants were annotated using ANNOVAR and the annotated variants were incorporated to the protein database to create custom variant protein database.

Proteomic searches were carried out using Proteome Discoverer 2.3 (Thermo Scientific, Bremen, Germany). Searches could also be carried out using freely available open source alternatives such as MaxQuant, MsFragger, MS-GF+, MyriMatch or OMSSA. Mass spectrometry raw files were obtained from the PRIDE archive ( PXD008222) and searched against the customized variant protein database using SEQUEST-HT search algorithm. Trypsin and LysC were set as proteolytic enzymes with a maximum missed cleavage of one. Carbamidomethylation of cysteine was set as a fixed modification, and acetylation of protein N-terminus and oxidation of methionine were set as variable modifications with a minimum peptide length of seven amino acids. The precursor mass tolerance was fixed as 10 ppm, and 0.05 Da for fragment ions. Mass spectrometry data were searched against the decoy database with 1% false discovery rate cut-off at the peptide level.

We have provided a dataset for users to test the tool. The test dataset was taken from the study SRR7418758 archived on the NCBI Sequence Read Repository (SRA) and aligned using human reference genome version 19 (hg19). Using samtools view command, reads mapped to chromosome 22 were extracted and converted to FASTQ files (paired-end). We have also provided chromosome 22 nucleotide sequences from hg19 and corresponding variant information from dbSNP database.

## Use cases

**Figure 2.**
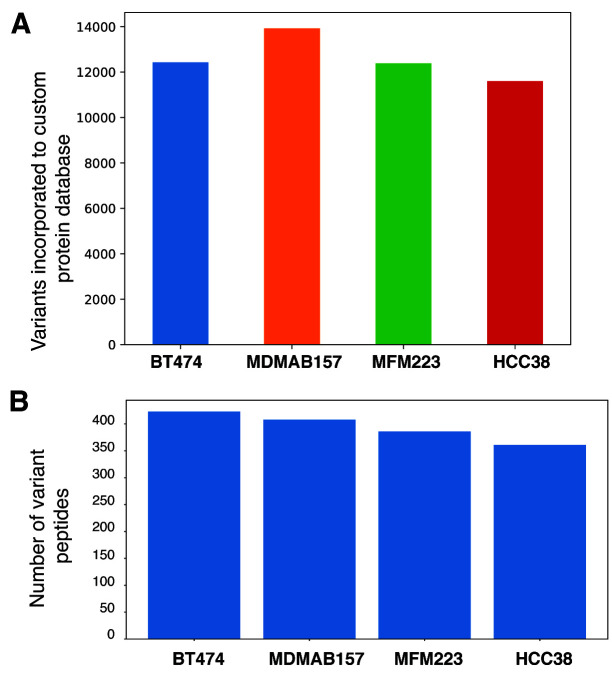
**A**) Number of nonsynonymous variants incorporated to protein database. **B**) Variant peptides identified across four cell lines (BT474, MDMAB157, MFM223 and HCC38) by searching mass spectrometry data against sample specific protein database.

As a test case, we analyzed exome ( Daemen *et al*., 2013) and proteome datasets ( Lawrence *et al.,* 2015) from breast cancer cell lines. We incorporated 12,429; 13,923; 12,386 and 11,600 non-synonymous SNPs from BT474 (accession number SRR925752), MDMAB157 (accession number SRR925788), MFM223 (accession number SRR925796) and HCC38 (accession number SRR925778) cells, respectively, to the protein database ( Figure 2A). These non-synonymous SNPs were incorporated to the reference protein database (Human RefSeq release 93) to create customized variant protein database. Mass spectrometry-based raw files were searched against their respective custom variant protein database, which resulted in identification of 423, 408, 386 and 361 variant peptides from BT474, MDMAB157, MFM223 and HCC38, cell line datasets ( Figure 2B). Interestingly, we observed mutant protein expression of Replication Timing Regulatory Factor 1 (RIF1) and Torsin-1A-interacting protein 1 (TOR1AIP1) across all four breast cancer cell lines. Mutant plectin (PLEC), marker Of Proliferation Ki-67 (MKI67), HEAT Repeat Containing 1 (HEATR1) and AHNAK nucleoprotein (AHNAK) were detected in three of the four breast cancer cell lines. These coding mutations have also been reported in other cancers The resultant variant peptide lists are available as *Underlying data* ( Kasaragod *et al.*, 2020a). The data generated from the tool demonstrates the usefulness and the ease of detection of variant peptides in an integrated omics analysis.

## Conclusions

CusVarDB generates customized variant protein database from NGS datasets. To our knowledge, this is the first tool which uses Linux Bash Shell to execute the NGS tools on a Windows OS. The tool provides additional feature of dry-run, making the commands highly customizable. The variant protein database generated by this tool is highly compatible for use as a reference protein database for analysis of variants at the proteomic level. CusVarDB currently allows incorporation of non-synonymous mutations. It does not allow incorporation of protein variations resulting from indels and frame-shifts. We developed a flexible tool with the intension of updating it in the future.

## Data availability

### Source data

Whole exome data from Daemen *et al.* (2013) on BioProject, Accession number PRJNA210427

Mass spectrometry proteome data from Lawrence *et al.* (2015) on PRIDE, Accession number PXD008222: https://identifiers.org/pride.project:PXD008222

Test dataset on SRA, Accession number SRR7418758: https://identifiers.org/insdc.sra:SRR7418758

### Underlying data

Zenodo: CusVarDB: A tool for building customized sample-specific variant protein database from Next-generation sequencing datasets. http://doi.org/10.5281/zenodo.4018694 ( Kasaragod *et al.*, 2020a)

This project contains the following underlying data:

- Gowda_CusVarDB_Supplementary_table1.xlsx (This table contains the resultant variant peptides along with the wild-type peptides from BT474, MDMAB157, MFM223, and HCC38 datasets. Along with mutant peptides, this section also provides additional information such as peptide-spectrum match [PSM], Protein accession, cross-correlation value from the search [Xcorr] and retention time [RT])
- Gowda_CusVarDB_Supplementary_table2.xlsx (This table provides the complete details of the resultant peptides. Here the mutant and corresponding wild-type peptides are mentioned in different sheets. For a given mutant peptide its wild-type peptide and corresponding information can be mapped using the VLOOKUP function in Excel by keeping column A [Sl.No] as lookup parameter.)
- Gowda_CusVarDB_Supplementary_table3.xlsx (This table briefs about the variants which are already reported in other cancers.)

Data are available under the terms of the Creative Commons Attribution 4.0 International license (CC-BY 4.0).

## Software availability

Software available from: https://sourceforge.net/projects/cusvardb/ and http://bioinfo-tools.com/Downloads/CusVarDB/V1.0.0/

Source code available from: https://github.com/sandeepkasaragod/CusVarDB

Archived source code at time of publication: https://doi.org/10.5281/zenodo.3780645 ( Kasaragod *et al.*, 2020b)

License: Creative Commons Attribution 4.0 International license (CC-BY 4.0).
