# Supplementary material for: CusVarDB: A tool for building customized sample-specific variant protein database from next-generation sequencing datasets
Source: F1000Res. 2020 Nov 16;9:344. Originally published 2020 May 11. [Version 2] doi: 10.12688/f1000research.23214.2 (PMC7684676; doi:10.12688/f1000research.23214.2)
Supplement: Peer review history

# Peer review: Reviewer response for version 2

**DOI:** 10.5256/f1000research.30424.r74962

**Published:** 2020-12-02

**Authors:** Jorge Duitama, Daniel Mahecha

**Affiliations:** 1 Systems and Computing Department, Universidad de Los Andes, Bogotá, Colombia

This manuscript by Kasagarod *et al.*, describes a GUI-based tool that executes a pipeline of Linux based NGS tools on a Windows OS, to create a variant protein database specific for a sample. This variant protein database can be further used to identify mutant proteins on mass spectrometry data from proteomics studies. 

 The manuscript claims that CusVarDB is a useful tool on the onco proteo-genomics and different from other tools that require BED or VCF files as input, while it works with FASTA and FASTQ files as an input. As a use case, it provides examples of its use in genomics and proteomics data from four cancer cell lines.

 Unfortunately, in my opinion there are several technical and conceptual issues related to this work:

1. Regarding decisions on technological architecture, it is not clear which is the advantage of building an application on technology that only runs on windows to run programs that run mostly on Linux. As a consequence, I could not install and execute the software successfully. The manuscript claims that VCF files are difficult to generate because: “Executing the preprocessing steps requires knowledge of computation and also requires multiple tools to generate the output.”. However, there are different commercial and open source solutions to analyze sequencing reads and generate VCF files. As an example, my research group maintains the Next-Generation Sequencing Experience Platform, which is able to make this analysis with a graphical interface on any operative system. Other well known web based solutions are Galaxy or CyVerse. For variants interpretation, functionalities of CusVarDB should be compared with those offered by well known platforms such as VEP.
2. One common issue with wrapping tools for other software such as GATK and bwa is that the software tools are evolving and hence the wrapping software quickly becomes outdated. Regarding this issue, the manuscript does not describe if a maintenance plan is planned for this tool, including the way different enclosed tools will be updated.
3. In the section “use cases”, the manuscript describes one single use case in which alternative proteins are identified for four samples. It is not clear which parameters were used to run the mapping and variant calling tools, and especially to filter variants. It is not clear if the reported variants are the complete set of variants after this process. The false positive rate of the proposed analysis pipeline should be estimated and compared with other pipelines to identify variants in cancer samples. Moreover, it is not clear if tumor and normal tissue was considered to increase the reliability of the reported variants. If the section is called “use cases”, then more use cases should be included.
4. From the description of the use case, it looks like both genomics and proteomics data is required to operate CusVarDB. Obtaining both data types can be too expensive for real clinical settings. The manuscript should clearly state if both data types are required or what can be done if only DNA sequencing data is available. 
5. The conclusions state that the variant protein database generated by this tool is highly compatible for use as a reference protein database for analysis of variants at the proteomic level. However, from the manuscript and the manual it is not clear what format the output variant protein database has and what information does it include, and whether the output format is compatible with commonly used proteomics tools. If the database is relational, an entity relationship model should be provided and instructions on how to install and use the database should be provided and maintained as part of the documentation.

Are the conclusions about the tool and its performance adequately supported by the findings presented in the article?

Partly

Is the rationale for developing the new software tool clearly explained?

Partly

Is the description of the software tool technically sound?

Partly

Are sufficient details of the code, methods and analysis (if applicable) provided to allow replication of the software development and its use by others?

No

Is sufficient information provided to allow interpretation of the expected output datasets and any results generated using the tool?

No

Reviewer Expertise:

Bioinformatics, applied algorithms, software development, population genomics

We confirm that we have read this submission and believe that we have an appropriate level of expertise to state that we do not consider it to be of an acceptable scientific standard, for reasons outlined above.

# Peer review: Reviewer response for version 2

**DOI:** 10.5256/f1000research.30424.r74866

**Published:** 2020-11-23

**Authors:** Suresh Mathivanan

**Affiliations:** 1 Department of Biochemistry and Genetics, La Trobe Institute for Molecular Science, La Trobe University, Melbourne, Vic, Australia

All issues addressed.

Are the conclusions about the tool and its performance adequately supported by the findings presented in the article?

Yes

Is the rationale for developing the new software tool clearly explained?

Yes

Is the description of the software tool technically sound?

Yes

Are sufficient details of the code, methods and analysis (if applicable) provided to allow replication of the software development and its use by others?

Yes

Is sufficient information provided to allow interpretation of the expected output datasets and any results generated using the tool?

Yes

Reviewer Expertise:

Cancer proteomics and bioinformatics

I confirm that I have read this submission and believe that I have an appropriate level of expertise to confirm that it is of an acceptable scientific standard.

# Peer review: Reviewer response for version 2

**DOI:** 10.5256/f1000research.30424.r74867

**Published:** 2020-11-16

**Authors:** Richard Kumaran Kandasamy

**Affiliations:** 1 Department of Clinical and Molecular Medicine, Norwegian University of Science and Technology, Trondheim, Norway

The authors have addressed the minor issues and I recommend accepting the article for indexing.

Are the conclusions about the tool and its performance adequately supported by the findings presented in the article?

Yes

Is the rationale for developing the new software tool clearly explained?

Yes

Is the description of the software tool technically sound?

Yes

Are sufficient details of the code, methods and analysis (if applicable) provided to allow replication of the software development and its use by others?

Yes

Is sufficient information provided to allow interpretation of the expected output datasets and any results generated using the tool?

Partly

Reviewer Expertise:

My research expertise include proteomics, proteogenomics, bioinformatics and systems immunology.

I confirm that I have read this submission and believe that I have an appropriate level of expertise to confirm that it is of an acceptable scientific standard.

# Peer review: Reviewer response for version 1

**DOI:** 10.5256/f1000research.25627.r63338

**Published:** 2020-08-26

**Authors:** Richard Kumaran Kandasamy

**Affiliations:** 1 Department of Clinical and Molecular Medicine, Norwegian University of Science and Technology, Trondheim, Norway

The manuscript by Kasaragod *et al.,* describes a software application for developing sample-specific protein sequence database that takes into account the protein-coding variants, for interpretation of mass spectrometry data to see what portion of the variants are seen at the protein expression level. This is an important aspect in exemplifying the use of genomic data on proteomics datasets associated with cancer samples.

 The software is made available for download and enough documentation has been provided for potential users.

 The authors use this data set to identify protein-level evidence for expression of variants in four different cancel cell lines.

 The manuscript can be accepted for indexing. I have one important point that I would like the authors to address:

 While the findings are potentially interesting, it would be nice if the authors pick a few important examples specific to these four cell lines.

 This would give the users a better appreciation of the biological insights given by this application.

Are the conclusions about the tool and its performance adequately supported by the findings presented in the article?

Yes

Is the rationale for developing the new software tool clearly explained?

Yes

Is the description of the software tool technically sound?

Yes

Are sufficient details of the code, methods and analysis (if applicable) provided to allow replication of the software development and its use by others?

Yes

Is sufficient information provided to allow interpretation of the expected output datasets and any results generated using the tool?

Partly

Reviewer Expertise:

My research expertise include proteomics, proteogenomics, bioinformatics and systems immunology.

I confirm that I have read this submission and believe that I have an appropriate level of expertise to confirm that it is of an acceptable scientific standard, however I have significant reservations, as outlined above.

# Peer review: Reviewer response for version 1

**DOI:** 10.5256/f1000research.25627.r63336

**Published:** 2020-07-23

**Authors:** Suresh Mathivanan

**Affiliations:** 1 Department of Biochemistry and Genetics, La Trobe Institute for Molecular Science, La Trobe University, Melbourne, Vic, Australia

This is a manuscript that describes an important tool. 

1. The authors may need to discuss other proteogenomic tools similar to this and provide the advantages. If such tools don't exist, perhaps make a strong case. Zhang lab tools can also be discussed with references.
2. Can the authors also discuss and refer previous attempts on onco-proteogenomics? One of the first paper on onco-proteogenomics came in 2012: Mathivanan, S., Ji, H., Tauro, B.J., Chen, Y.S. and Simpson, R.J. (2012) Identifying mutated proteins secreted by colon cancer cell lines using mass spectrometry. ***Journal of Proteomics***. 76, 141-9 ^1^.
3. Does CurVardb provide only non-synonymous point mutations? What about frame shit mutations? Is that also provided or perhaps will be added in next version.

Are the conclusions about the tool and its performance adequately supported by the findings presented in the article?

Yes

Is the rationale for developing the new software tool clearly explained?

Yes

Is the description of the software tool technically sound?

Yes

Are sufficient details of the code, methods and analysis (if applicable) provided to allow replication of the software development and its use by others?

Yes

Is sufficient information provided to allow interpretation of the expected output datasets and any results generated using the tool?

Yes

Reviewer Expertise:

Cancer proteomics and bioinformatics

I confirm that I have read this submission and believe that I have an appropriate level of expertise to confirm that it is of an acceptable scientific standard, however I have significant reservations, as outlined above.

## References

[1] : Identifying mutated proteins secreted by colon cancer cell lines using mass spectrometry. *J Proteomics*.2012;76 Spec No.: 10.1016/j.jprot.2012.06.031 141-9 10.1016/j.jprot.2012.06.031 22796352
